# Molecular classification of tissue from a transformed non-Hogkin’s lymphoma case with unexpected long-time remission

**DOI:** 10.1186/s40164-016-0063-0

**Published:** 2017-01-11

**Authors:** Julie Støve Bødker, Marianne Tang Severinsen, Tarec Christoffer El-Galaly, Rasmus Froberg Brøndum, Maria Bach Laursen, Steffen Falgreen, Mette Nyegaard, Alexander Schmitz, Lasse Hjort Jakobsen, Anna Amanda Schönherz, Hanne Due, Linn Reinholdt, Martin Bøgsted, Karen Dybkær, Hans Erik Johnsen

**Affiliations:** 1 Department of Hematology, Aalborg University Hospital, Aalborg, Denmark; 2 Clinical Cancer Research Center, Aalborg University Hospital, Aalborg, Denmark; 3 Department of Clinical Medicine, Aalborg University, Sdr. Skovvej 15, 9000 Aalborg, Denmark

**Keywords:** Transformed lymphoma, Long-time remission, Molecular classification, Rituximab palliation, Drug resistance prediction

## Abstract

**Background:**

The concept of precision medicine in cancer includes individual molecular studies to predict clinical outcomes. In the present N = 1 case we retrospectively have analysed lymphoma tissue by exome sequencing and global gene expression in a patient with unexpected long-term remission following relaps. The goals were to phenotype the diagnostic and relapsed lymphoma tissue and evaluate its pattern. Furthermore, to identify mutations available for targeted therapy and expression of genes to predict specific drug effects by resistance gene signatures (REGS) for R-CHOP as described at http://www.hemaclass.org. We expected that such a study could generate therapeutic information and a frame for future individual evaluation of molecular resistance detected at clinical relapse.

**Case presentation:**

The patient was diagnosed with a transformed high-grade non-Hodgkin lymphoma stage III and treated with conventional R-CHOP [rituximab (R), cyclophosphamide (C), doxorubicin (H), vincristine (O) and prednisone (P)]. Unfortunately, she suffered from severe toxicity but recovered during the following 6 months’ remission until biopsy-verified relapse. The patient refused second-line combination chemotherapy, but accepted 3 months’ palliation with R and chlorambucil. Unexpectedly, she obtained continuous complete remission and is at present >9 years after primary diagnosis. Molecular studies and data evaluation by principal component analysis, mutation screening and copy number variations of the primary and relapsed tumor, identified a pattern of branched lymphoma evolution, most likely diverging from an in situ follicular lymphoma. Accordingly, the primary diagnosed transformed lymphoma was classified as a diffuse large B cell lymphoma (DLBCL) of the GCB/centrocytic subtype by “cell of origin BAGS” assignment and R sensitive and C, H, O and P resistant by “drug specific REGS” assignment. The relapsed DLBCL was classified as NC/memory subtype and R, C, H sensitive but O and P resistant.

**Conclusions:**

Thorough analysis of the tumor DNA and RNA documented a branched evolution of the two clinical diagnosed tFL, most likely transformed from an unknown in situ lymphoma. Classification of the malignant tissue for drug-specific resistance did not explain the unexpected long-term remission and potential cure. However, it is tempting to consider the anti-CD20 immunotherapy as the curative intervention in the two independent tumors of this case.

**Electronic supplementary material:**

The online version of this article (doi:10.1186/s40164-016-0063-0) contains supplementary material, which is available to authorized users.

## Background

Follicular lymphoma (FL) is a low-grade non-Hodgkin’s type lymphoma with a median survival of 8–10 years [1, 2]. It can evolve into the more aggressive histology of transformed FL (tFL) [3], resembling diffuse large B-cell lymphoma (DLBCL), but share a distinct gene expression profile and immunophenotype with the primary FL [4–8]. This transformation occurs by the expansion of one or more subclones with loss of the follicular growth pattern resulting in a rapidly progressive clinical course refractory to treatment and with inferior prognosis. To distinguish between progression and transformation of FL a biopsy for histological examination is needed in case of symptoms and CT verified tumor progression. The histological verified presence of both follicular and diffuse architecture in the initial diagnostic biopsy represents a finding that implies early transformation of FL [2, 3]. The survival post-transformation ranges between median 7–20 months [9, 10].

Similar to progression, the transformation is a clonal evolution from an ancestral clone that initially arises from a common normal cell of origin (COO) through linear or branched outgrowth of an existing subclone, deregulated by random genetic or epigenetic hits.

Our knowledge of genetic aberrations in FL has dramatically increased over the last few years, and recent comprehensive studies by exome sequencing has identified the mutational landscape and the genetic changes that contribute to the step wise tumor progression, including transformation to DLBCL [4–8, 11–18].

Here we report a unique, biopsy and histology verified case of primary tFL with relapse and long-term remission following palliation therapy. As molecular studies of such unexpected outcome may be informative, we decided to study the two malignant tissue biopsies and a germ line DNA sample to thorough analysis with the aim to identify differences in an attempt to understand and explain the clinical outcome.

The case will also illustrate the molecular technologies, knowledge and competences that are increasingly available to challenge the “one size fits all” approach to early clinical phase I–II drug trials in relapsed patients and replace this with a predictive strategy. We envisage that therapies will be stratified to reflect disease heterogeneity, in a departure from the current use of non-precise chemotherapeutic agents and allow for the development of new taxonomy and companion diagnostics—a clear need in future clinical care.

## Case presentation

Early August, 2007, a previously healthy 74 year-old woman was referred to the Department of Hematology at Aalborg University Hospital with a 3-month history of weight loss (5%) and abdominal pain. A computed tomography (CT) was performed as part of the initial diagnostic work-up and revealed a bulky retroperitoneal tumor measuring 13.5 × 12 × 7.5 cm. Pathologically enlarged lymph nodes were also detected in all supra- and infra-diaphragmatic stations and the spleen was enlarged (Additional file 1: Figure S1A, July, 2007). Furthermore, she had bilateral hydronephrosis.

A tumor biopsy from an axillary lymph node was performed. Morphological examinations revealed a picture typical for diffuse large B-cell lymphoma (DLBCL), but with nodular areas consistent with an underlying follicular lymphoma. The cells were monoclonal IgM^+^, kappa^+^, CD20^+^, CD19^+^, CD10^+^, CD38^+^, CD79A^+^, BCL2^+^ and BCL6^+^, CD30^−^ with a high Ki67-estimated sproliferation rate. On the basis of these findings a diagnosis of DLBCL transformed from follicular lymphoma was made. The routine bone marrow biopsy and aspiration was clear for signs of lymphoma infiltration. The disease was categorized as stage III according to the Ann Arbor classification based on the extensive nodal involvement on both sides of the diaphragm.

A combined modality treatment with six cycles of R-CHOP (Rituximab, cyclophosphamide, doxorubicin (hydroxydaunomycin), vincristine (oncovin) and prednisolon) followed by consolidating radiotherapy against the abdominal bulk tumor was planned. Unfortunately, her treatment was complicated by severe infectious episodes including septicemia. After the final R-CHOP cycle, she had lost an additional 10 kg and was in a clinical poor performance status. An 18F-FDG positron emission tomography/CT (PET/CT) performed at this point showed a residual PET-negative tumor mass surrounding the aorta (Additional file 1: Figure S1B, January, 2008). As a result of her poor condition she entered post-therapy follow-up without receiving the planned radiotherapy.

A routine PET/CT study performed after three months of follow-up confirmed continues remission (Additional file 1: Figure S1C, March, 2008). However, a routine PET/CT study performed 6 months into the follow-up period revealed clear progression of the retroperitoneal tumor in terms of increased size and FDG-uptake (SUV_max_ 11.2) (Additional file 1: Figure S1D, July, 2008). A tumor biopsy directed from an enlarged cervical lymph node revealed a morphological and immunohistochemical picture similar to that seen in the primary diagnostic biopsy (see above)—thus being consistent with relapse of DLBCL transformed from a follicular component.

In light of the severe complications during the primary R-CHOP therapy, she declined intensive chemotherapy and specifically asked for a palliative approach. She therefore received four doses of rituximab given every 3 weeks combined with continues oral chlorambucil (Leukeran^®^) at a dose of 4 mg per day as maintenance. Chlorambucil was stopped after 6 month due to side effects. At this point she was unwilling to accept any further lymphoma directed therapies or investigations but accepted clinical follow up including a PET/CT scan documenting remission.

Surprisingly, she slowly recovered and regained weight over the following months and a control PET/CT showed remission (Additional file 1: Figure S1E, July, 2010). At the last follow-up visit August 2016, 9 years after the initial diagnosis, she was still in good health without lymphoma-related symptoms or findings.

## Method section

### The aim, design and setting of the study

Based on the unexpected long-term remission of a tFL patient on palliative therapy for her first relapse, we obtained informed consent to perform a thorough molecular analysis on her primary and relapsed tumor. The idea was to explore den molecular background in her tumor samples in search for an explanation for her cure following palliation with rituximab (Mabthera^®^) and chlorambucil (Leukeran^®^).

Additional malignant tissue were available and included, from NHL patients at time of diagnosis before treatment and diagnosis were evaluated according to the Revised European-American Lymphoma Classification [1] and confirmed by two expert hematopathologists at Aalborg University Hospital. Included were patients who had tissue stored in the “Diagnostic Biobank, Department of Hematology, Aalborg” and clinical data, staging, therapy and outcome registered in the National Clinical Quality Database for lymphoma, as approved by the local ethical committee (RetroGene, N-20140099).

### Gene expression profiling (GEP)

RNA from 50 lymphoma samples FL (n = 7), tFL (n = 2; the case) and DLBCL (n = 41) was purified, labeled and hybridized to Affymetrix GeneChip Human Genome U133 Plus 2.0 Arrays and.CEL files generated as described [18] and presented in Additional file 2: Table S1. All were diagnostic samples taken before initiation of treatment. The relapse tFL sample (H385) being the exception, as the patients received R-CHOP treatment for her primary tumor (H302).

### Resistance gene signatures (REGS) for drug response

The effect of the drugs cyclophosphamide (C), doxorubicin (H), vincristine (O) and rituximab (R) on viable proliferating B-cell lines was measured by an in vitro drug screen strategy as recently described by our group [19–24]. Sensitivity or resistance towards individual drugs can be predicted by the REGS assignment of probability, which ranges from zero to one, respectively. A website (http://www.hemaClass.org) [25] has been generated to grant access to the classification and prediction tools to all researchers by easy upload of .CEL-file data. The.CEL files from the patient case tumors were analyzed and as build-in reference, our collection of lymphoma samples (RetroGene) performed at our laboratory was chosen in the RMA pre-processing normalization step. We chose the following classification systems: BAGS, R, C, H, O, P and R-CHOP, with the following ranges of non classified/intermediate groups: 0.1–0.9; 0.38–0.54; 0.33–0.55; 0.14–0.9; 0.46–0.62; 0.09–0.93, respectively. The results are downloaded and displayed in Table 3.

### DNA purification

DNA from the patient’s two tumor samples was purified as described [26]. The patient’s saliva sample was collected using the Oragene DNA Self-Collection Kit (OG-500) and the DNA purified from non-involved mucosa cells using the Oragene DNA purifier (OG-L2P) following manufactures instructions in protocol PD-PR-006 Issue 3.2 (DNA Genotek Inc, Ottawa, Ontario, Canada).

### Global copy number variation (CNV)

The purified DNA was labeled and hybridized to Affymetrix SNP6 arrays as described [26]. The .CEL files, generated through AGCC after scanning the arrays, were imported to Partek Genomics Suite Software v. 6.6 (6.14.0828) using interrogating probes only with a pre-background adjustment for GC content and probe sequence. Probe sets wire summarized using allele specific summarization, and normalized against the human Hapmap genome. Amplification and deletions were detected through genomic copy number segmentation using standard settings, in brief: minimum 10 genomic markers, a P value threshold of 0.001, and a signal to noise ratio of 0.3. The diploid copy number range was set to 1.7–2.3.

### Exome sequencing and analysis

From the patient’s saliva, primary and relapse tumor samples, standard sequence libraries for studies of point mutations were created from 100 ng of DNA from each sample, following exome capture using the Agilent SureSelect Human All Exon 50 Mb system (Agilent Technologies). Sequencing was performed on a HighSeq 2000 (Illumina, Hinxton, UK) using 76-bp paired-end reads, as described [27, 28].

Raw reads from the sequencer were processed following the Genome Analysis Toolkit (GATK) best practice guidelines [29, 30]. Reads were aligned to the grch37 assembly of the human genome with BWA v0.7.12 [31]. Aligned reads were sorted, converted to BAM format and had PCR duplicates marked using Picard v2.0.1 (http://broadinstitute.github.io/picard/). Sequence realignment around INDELs and adjustment of quality scores was done using GATK v.3.5.0 [32]. Discovery of somatic variants in both primary and relapse tumors was performed using MuTect2 [33] incorporating information from dbSNP v138 [34] and COSMICv75 [35]. Somatic variants that passed quality filters were annotated using Oncotator v.1.8.0.0 [36] and finally PHIAL v1.0 [37] was used to score and rank somatic mutations according to clinical relevance and to identify potential targeted therapy for the analyzed patient.

### Datasets

The micro array data are deposited at Gene Expression Omnibus in project GSE86622, see Additional file 2: Table S1. The exome sequencing data from the tFL patient samples: non-involved mucosa cells, the lymphoma diagnostic sample and the sample following clinical relaps, are available through the European Genome Phenomena Archive at the European Bioinformatics Institute under accession number EGAD00001002707.

### Statistical analysis

All statistical analyses were performed using R (The R Development Core Team, 2013) version 3.0.1 [38–41].

## Results

In the present N = 1 case we have analysed the diagnostic and relapsed lymphoma tissue DNA and RNA by exome sequencing and microarray expression respectively. The primary goal was to phenotype the lymphoma tissues and evaluates its evolution pattern. The secondary goals were in a therapeutic context to identify specific mutations available for targeted therapy and expression of genes to predict specific drug effects of R-CHOP. It is expected that we could generate information useful in this specific and unexpected case.

### Principal component analysis of DLBCL, FL and the case samples

**Fig. 1:**
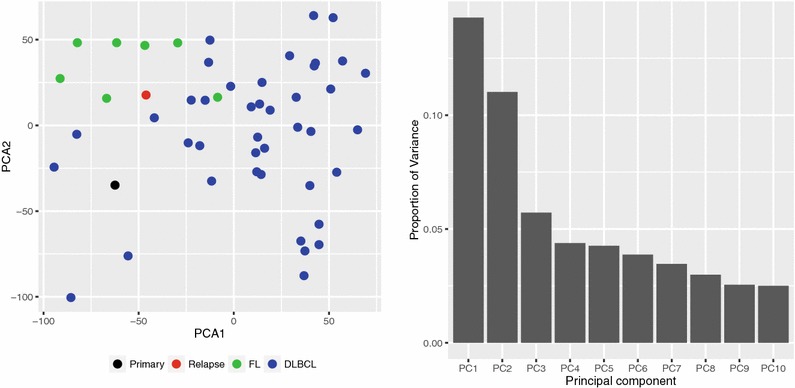
Principal component analysis (PCA) of the tFL case compared to FL and DLBCL samples. A principal component (PC) analysis on GEP from 7 FL, 41 DLBCL samples and the patient’s primary and relapse tumors was performed. All probe sets for all samples were included in the PC analysis. **a** The two diagnostic entities, FL and DLBCL, segregated into distinct clusters in the PC analysis, with the primary and relapse tumor samples located at the edges of the FL and DLBCL groups, respectively. **b** The proportion of variance in the 10 first PC’s are displayed

The gene expression profiles of the primary and relapse samples from our patient case were analyzed together with 7 FL and 41 DLBCL cases from our clinic in a principal component analysis (PCA) with the results as illustrated in Fig. 1. The cases formed two distinct groups, where the diagnostic and relapse tFL samples were different, in close proximity to the FL and DLBCL groups, respectively.

### Mutation identification by exome sequencing of the case samples

We performed exome sequencing on the primary and the relapse tumor and identified somatic changes in 687 SNPs and INDELs compared to the constitutional DNA obtained from the patient’s saliva. The primary (H302) and relapse (H385) tumors contained 329 and 358 somatic tumor specific changes, respectively, with an overlap of 127, as shown in Table 1. A thorough review of clinically relevant mutated genes, two (*EZH2* and *DNMT3A*) were unique for the primary tumor and two were unique for the relapse tumor (*FBXW7* and *FIP1L1*). Three of the genes were found in both tumors (*NOTCH2*, *TP53* and *EP300*), however, *NOTCH2* were present at different allelic fractions in the two tumors (Table 2). Of the 37 recurrent gene mutations described by Pasqualucci [8] we recognized mutations in *BCL2, HIST1H1E, EP300, TP53 and STAT6* in both tumors, *EZH2* in the primary and *KDM6B* in the relapsed tissue. Overall, the mutation pattern recognized suggests a branched evolution from an unknown common mutated ancestor through the independent acquisition of distinct genetic lesions.

**Table 1:** Classification of variants in primary and relapsed tumor

Variant	Primary	Relapse	Shared
3’UTR	4	10	4
5’Flank	1	1	0
5’UTR	2	2	1
Frame_Shift_Del	0	3	0
Frame_Shift_Ins	2	1	0
IGR	48	42	12
In_Frame_Del	0	3	0
Intron	109	124	42
lincRNA	4	8	2
Missense_Mutation	110	94	46
Nonsense_Mutation	3	6	2
RNA	18	20	8
Silent	23	37	7
Splice_Site	5	7	3
Total	329	358	127

**Table 2:** Clinically relevant variants detected by PHIAL

		Coverage		Allelic fraction	
Gene	Variant	Primary	Relapse	Primary	Relapse
TP53	Missense_Mutation	35	37	0.66	0.62
NOTCH2	Nonsense_Mutation	24	26	0.58	0.27
EP300	Missense_Mutation	55	63	0.31	0.33
DNMT3A	Splice_Site	52	NA	0.08	NA
EZH2	Missense_Mutation	37	NA	0.3	NA
FBWX7	Missense_Mutation	NA	64	NA	0.38
FIP1L1	Splice_Site	NA	59	NA	0.37

**Fig. 2:**
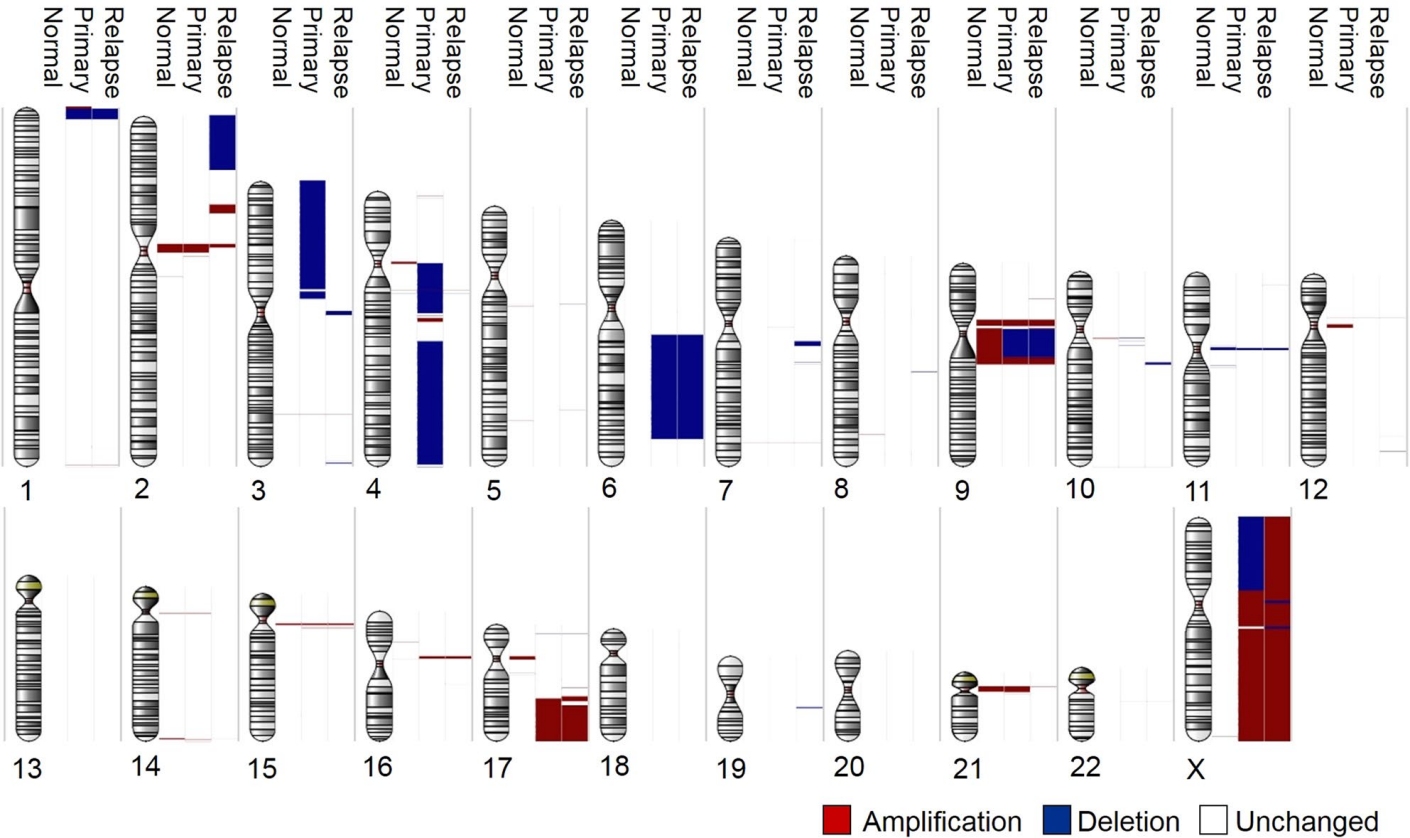
Copy number analysis of diagnostic and relapsed tFL tissue. Copy number variations above 100 kb in all chromosomes are displayed for the saliva sample, the primary and the relapse tumors from the tFL patient case. The image was generated through Partek™ Genomics Suite Software

This was also visualized by global SNP6-microarray analysis for copy number variations (CNV) above 100 kb as illustrated in Fig. 2. The diagnostic and relapse samples had various chromosomal regions with identical CNV, illustrated for chromosomes 1, 6, 9, 16, 17 and X. However, many CNVs were unique to the primary tumor—see chromosomes 3, 4, 7, 10, 17 and X, or unique to the relapsed tissue seen in chromosomes 2, 3, 4, 5, 7, 8, 10, 11, 12, 16, 18, 19.

In summary, the diagnostic and relapsed tFL harbor unique mutations and CNVs documenting that the tumors have a common genetic background and the tFL case is a consequence of branched and not direct linear clonal selection and evolution.

### Estimation of drug sensitivity for the case samples

The primary and relapse samples were assigned a cell of origin (COO) subtype as described [18] resistance estimate for R-CHOP by REGS [19–21] classifiers following assignment in http://www.hemaClass.org [25]. This tool provides an easy interface for one-by-one microarray based classification based on our preclinical models for BAGS and REGS. By individual REGS assignment, the primary tumor was predicted to be resistant to the drugs C, H, O and P, but sensitive towards R. In contrast, the relapse tumor had changed drug sensitivity for the alkylating agents C but still resistant towards O and P as well as the very high sensitive towards R, as shown in Table 3.

**Table 3:** Probability of resistance and predicted R-class from http://www.Hemaclass.org

Predicted classification	Primary tumor (H302)	Relapse tumor (H385)
Probability of BAGS	0.595	0.363
BAGS class	Centrocyte	Memory
Probability of ABC	1.19E + 09	1.09E − 01
ABC-GCB-NC class	GCB	NC
Probability of R	0.034	0.049
R class	Sensitive	Sensitive
Probability of C	0.662	0.225
C class	Resistant	Sensitive
Probability of H	0.963	0.041
H class	Resistant	Sensitive
Probability of O	0.750	0.639
O class	Resistant	Resistant
Probability of Dex	0.023	0.037
Dex class	Resistant	Resistant
Probability combined	0.993	0.022
Combined class	Resistant	Sensitive

The probability of resistance towards cyclophosphamide (C), doxorubicin, (H), vincristine (O), and rituximab (R) determined by applying the REGS classification (Dybkaer et al. [18] and Laursen et al. [19] in preparation) onto .CEL files from Affymetrix U133plus2.0 arrays of the indicated samples through http://www.hemaclass.org. A probability close to 1 predicts resistance, whereas a value close to 0, predicts sensitivity to the indicated drug

*NC* not classified

In summary, the gene signature based subtyping of the two tFL tissue support that they were different although with important overlaps. From a functional perspective both tissues are R sensitive, indicating that the patient was cured by the targeted anti-CD20 therapy.

## Discussion

“N of 1” case studies require prospective collection of individual tissue into biobanks and storage of molecular data from standardized tissue analysis. Here we describe a unique patient who was “cured” for tFL and we present data that identify the differential pattern of genetic evolution and furthermore classify the lymphomas by resistance estimates for the specific drugs in question.

The tFL presented in this case was advanced stage at diagnosis and a poor outcome was expected [9]. Even if such cases are rare, we believe that the knowledge from our work will be useful if we can collect and assemble such data across several cases [42]. In the era of precision medicine, the design of various types of medical “data commons” may allow us to compare individual cases. It is our hope that other research groups will perform similar extensive molecular analysis of tFL cases with long-term remission in order to improve understanding of pathogenesis and individual treatment response.

### Documentation of lymphoma evolution

Sequencing the malignant genome has opened new knowledge to the complexity of malignant lymphoma cell genetics, selection and Darwinian evolution [43, 44]. Multiple studies have analysed paired biopsies from indolent follicular lymphoma and its transformation for differential genetic aberrations associated to the clinical progression. A common finding is the premalignant *IGH*-*BCL2* hybrid transcript expression and several key genetic events that seem to drive the process of progression [5, 6, 8]. In parallel, comparative analysis can also identify the genotypic background and, as in our case, document patterns of branched or linear lymphoma evolution. From published data, we have a long list of recurrent driver mutations from functional screening that illustrates the complexity and how heterogeneous the tFL genomes actually are.

In the present case, recurrent somatic mutations of *BCL2, NOTCH2*, *TP53* and *EP300 were identified in* the primary and relapsed tumor, which indicates a common progenitor cell, most likely a transformation from an unknown in situ or premalignant follicular lymphoma [45, 46]. The branched evolution was supported by the presence of gene mutations unique to the primary tFL tumour, e.g. the recurrent genes, *EZH2* and *DNMT3A* that were mutated in 30 and 8% of the reads, but not mutated in the reads from the relapse tFL tumour. On the contrary the relapsed tumor had several specific mutations e.g. in *FBXW7* and *FIP1L1*.

In summary, the data (Figs. 1, 2 and Tables 1, 2) confirms that each of the individual tFL in our patient case has unique genomic profiling. The precision medicine concept argues that genetic heterogeneity is a key-factor for therapeutic failure. This limitation is broadly recognized and represents a considerable challenge, technically and bioinformatically. Understanding the genetic diversity and how it changes in response to interventions, will require deep sequencing and analysis of the genomes of highly selected single cells [47, 48].

### Impact of gene signature classification of COO and drug resistance

Despite the enormous resources spent on developing molecular based cancer classification systems, most of these are still not available in clinical practice. To allow implementation and fast validation of our recent findings in DLBCL [18–21], we have developed an easily accessible web application that permits other users to assign ABC/GCB, B-cell associated gene signature (BAGS) as well as drug specific resistance gene signature (REGS) on their own datasets. The website called hemaClass.org [25] is a new prospect for easy individual subtyping of malignant B cell diseases; in particular for DLBCL and myeloma.

In summary, the primary diagnosed tFL was classified as a diffuse large B cell lymphoma (DLBCL) of the GCB/centrocytic subtype by “cell of origin BAGS” assignment and R-CHOP resistant by “drug specific REGS” assignment. The relapsed DLBCL was classified as NC/memory subtype and R-CHOP sensitive in support of the branched evolution. Monitoring such functional variations following treatment may identify the mechanisms of molecular drug resistance resulting in clinical relapse.

### Limitations of the study

This case study may be important for future diagnostic phenotyping and implementation of individual targeted drug or specific predictive therapy, however it involves a range of clinical, biological, and statistical limitations to be considered.

*First*, we cannot trust the conventional classification of poor prognosis associated with tFL, to make a clinical decision at the individual level. However, we expected this to be fulfilled by molecular profiling of drug specific sensitivity and resistance and mutations status for targeted therapy by designer drugs [49]. However, this strategy needs to be prospective validated by selected clinical end point, like level of remission, event free or overall survival. Such studies are ongoing, also in our center including all relapsed patients with haematological malignancies, to be used in the future evidence based strategy for individualized targeted and predicted therapy [49–53].

*Second*, the tissues analyzed are obtained from the biopsy of a single tumor focus, knowing that an entirely different profile might be seen from the biopsy of an adjacent lesion. The identification of the mutations within sub-clones is lost when DNA is extracted from the total cell population. This is most important if patient-and drug-specific genomic profiles are used for selecting therapeutic targets. This limitation represents a considerable challenge, technically and bioinformatically and will require deep sequencing and analysis of the genomes of single cells sorted by multiparametric flow cytometry.

*Third*, this case study has described a frame for future individual evaluation of clinical resistance detected at relapse. However, the individual REGS assignments given in Table 3 showed that the two tFL biopsies are R sensitive and indicate a clinical response to targeted anti-CD20 therapy for both tissues. However, we did not have a drug specific REGS predictor for the alkylating drug Chlorambucil, but the shift in sensitivity for the alkylating drug C may illustrate a potential sensitivity also to Chlorambucil therapy and a clinical impact in combination with R [54, 55]. Ongoing preclinical drug screens do include clinical available drugs and clinical relevant combinations.

*Fourth*, it has to be stressed that the clinical impact of REGS assignment is documented by retrospective analysis of thousands of patient sample data from several international clinical drug trials [20–23]. Therefore, we need to await ongoing prospective implementation trials validating the clinical impact of REGS assignment in clinical resistant haematological malignancies, before introducing our “second generation” companion diagnostics for malignant B cell diseases. However, the present case illustrates our research strategy for implementation of individualized care focused on patients with relapse or progression. The specific challenges in this area are the unexplained molecular drug resistance and the undocumented use of drug combinations transferred from the “one size fits all” approach. Key to address this challenge is an understanding of the molecular/genetic profiles of each tumour such that we can tailor therapy appropriately, generate improved and evidence based clinical outcomes, and make the most efficient use of healthcare resources. Predictive companion diagnostics will identify multidrug resistant patients that will be extensively characterized and screened for specific pathway and/or mutations, attempting to validate target therapy by small and limited number of patients [56].

## Conclusions

The present case of primary tFL was treated with conventional R-CHOP and suffered from severe side effects but obtained a 6 months remission. However, she relapsed after 6 months and accepted palliation with rituximab and chlorambucil for 6 months. Unexpectedly, she obtained a continuous complete remission, at present >9 years after primary diagnosis.

The retrospective analysis of the tumor tissue, documented the following key points:

- Genomic sequencing, CNV and gene expression defined a branched evolution of the two independent lymphomas, most likely transformed from an unknown lymphoma in situ.
- Classification for drug specific sensitivity and resistance propose the targeted anti-CD20 antibody therapy with the curative potential in the present case.

Together this case foresees a paradigm shift in the clinical treatment culture for relapsed patients with haematological malignancies—toward “personalised medicine” and “precision medicine” that need individual molecular work out [49–53].

## Additional files

**Additional file 1.** Diagnostic image examinations of the patient case. A) The computed tomography (CT) from the primary lymphoma diagnosis showed retroperitoneal bulk tumor and bilateral hydronephrosis (July 2007). B) A PET/CT was performed after six cycles of R-CHOP and identified a large abdominal residual tumor without FDG (January 2008). C) A routine scan was performed after three months of follow-up and confirmed continues remission (March 2008). D) A control scan was performed six months after therapy showing FDG accumulation in the retroperitoneal residual tumor as well as a palpable cervical tumor (July 2008). After four cycles of rituximab and continues chlorambucil treatment (total 6 months) a status scan was performed and showed a marked reduction of FDG-uptake in the abdominal bulk tumor (November 2008 - data not shown), and repeated as illustrated in E) consistent with continuous complete remission (July 2010) according to the revised response criteria for malignant lymphoma. The superficial process at abdomen in E) was a benign lipoma.

**Additional file 2.** Micro array data files. The Affymetrix .CEL files were deposited at the GEO website under GSE86622. In total, U133 plus 2.0 arrays were performed on 41 DLBCL, 2 tFL and 7 FL patient samples and 3 SNP6.0 arrays were performed on the saliva, primary and relapse tumors on the patient case. Clinical data are registered in the National Clinical Quality Database for Malignant Lymphoma (http://www.lymphoma.dk).

## Abbreviations

C: cyclophosphamide
H: doxorubicin
O: vincristine
P: prednisolon
R: rituximab
BAGS: B cell associated gene signature
REGS: drug resistence gene signature
DLBCL: diffuse large B-cell lymphoma
tFL: transformed follicular lymphoma
FL: follicular lymphoma
SNP: single-nucleotide polymorphism
INDEL: insertion or the deletion of bases in the DNA
CNV: copy number variation
COO: cell of origin
NHL: non Hodgkin lymphoma
