# Supplementary material for: Molecular classification of tissue from a transformed non-Hogkin’s lymphoma case with unexpected long-time remission
Source: Exp Hematol Oncol. 2017 Jan 11;6:3. doi: 10.1186/s40164-016-0063-0 (PMC5225590; doi:10.1186/s40164-016-0063-0)
Supplement: Supplementary file 1 — Additional file 1. Diagnostic image examinations of the patient case. A) The computed tomography (CT) from the primary lymphoma diagnosis showed retroperitoneal bulk tumor and bilateral hydronephrosis (July 2007). B) A PET/CT was performed after six cycles of R-CHOP and identified a large abdominal residual tumor without FDG (January 2008). C) A routine scan was performed after three months of follow-up and confirmed continues remission (March 2008). D) A control scan was performed six months after therapy showing FDG accumulation in the retroperitoneal residual tumor as well as a palpable cervical tumor (July 2008). After four cycles of rituximab and continues chlorambucil treatment (total 6 months) a status scan was performed and showed a marked reduction of FDG-uptake in the abdominal bulk tumor (November 2008 - data not shown), and repeated as illustrated in E) consistent with continuous complete remission (July 2010) according to the revised response criteria for malignant lymphoma. The superficial process at abdomen in E) was a benign lipoma. [file 40164_2016_63_MOESM1_ESM.pdf]

Additional file 1

Figure S1A-E.pdf

Diagnostic image examinations of the patient case

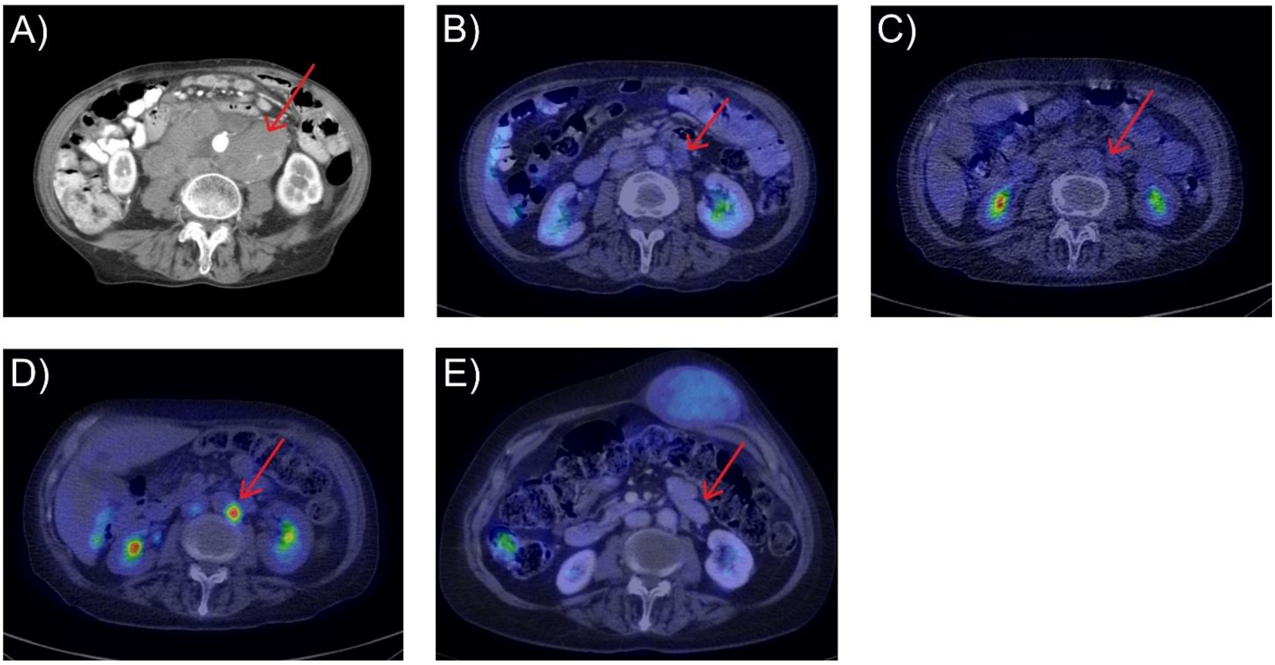

Figure Legend: A) The computed tomography (CT) from the primary lymphoma diagnosis showed retroperitoneal bulk tumor and bilateral hydronephrosis (July 2007). **B**) A PET/CT was performed after six cycles of R-CHOP and identified a large abdominal residual tumor without FDG (January 2008). **C**) A routine scan was performed after three months of follow-up and confirmed continues remission (March 2008). **D**) A control scan was performed six months after therapy showing FDG accumulation in the retroperitoneal residual tumor as well as a palpable cervical tumor (July 2008). After four cycles of rituximab and continues chlorambucil treatment (total 6 months) a status scan was performed and showed a marked reduction of FDG-uptake in the abdominal bulk tumor (November 2008 - data not shown), and repeated as illustrated in **E**) consistent with continuous complete remission (July 2010) according to the revised response criteria for malignant lymphoma. The superficial process at abdomen in **E**) was a benign lipoma.
