# Supplementary material for: Molecular classification of tissue from a transformed non-Hogkin’s lymphoma case with unexpected long-time remission
Source: Exp Hematol Oncol. 2017 Jan 11;6:3. doi: 10.1186/s40164-016-0063-0 (PMC5225590; doi:10.1186/s40164-016-0063-0)
Supplement: Supplementary file 2 — Additional file 2. Micro array data files. The Affymetrix .CEL files were deposited at the GEO website under GSE86622. In total, U133 plus 2.0 arrays were performed on 41 DLBCL, 2 tFL and 7 FL patient samples and 3 SNP6.0 arrays were performed on the saliva, primary and relapse tumors on the patient case. Clinical data are registered in the National Clinical Quality Database for Malignant Lymphoma (http://www.lymphoma.dk). [file 40164_2016_63_MOESM2_ESM.pdf]

Additional file 2

Table S1.pdf

Micro array data files

Patient	Disease	Array-type	GSM#
H12	DLBCL	U133+2	GSM2306879
H14	DLBCL	U133+2	GSM2306880
H2	DLBCL	U133+2	GSM2306881
H22	DLBCL	U133+2	GSM2306882
H284	DLBCL	U133+2	GSM2306883
H285	DLBCL	U133+2	GSM2306884
H295	DLBCL	U133+2	GSM2306885
H296	DLBCL	U133+2	GSM2306886
H297	DLBCL	U133+2	GSM2306887
H300	DLBCL	U133+2	GSM2306888
H318	DLBCL	U133+2	GSM2306889
H321	DLBCL	U133+2	GSM2306890
H330	DLBCL	U133+2	GSM2306891
H333	DLBCL	U133+2	GSM2306892
H344	DLBCL	U133+2	GSM2306893
H345	DLBCL	U133+2	GSM2306894
H371	DLBCL	U133+2	GSM2306895
H378	DLBCL	U133+2	GSM2306896
H380	DLBCL	U133+2	GSM2306897
H437	DLBCL	U133+2	GSM2306898
H438	DLBCL	U133+2	GSM2306899
H439	DLBCL	U133+2	GSM2306900
H440	DLBCL	U133+2	GSM2306901
H441	DLBCL	U133+2	GSM2306902
H442	DLBCL	U133+2	GSM2306903
H443	DLBCL	U133+2	GSM2306904
H444	DLBCL	U133+2	GSM2306905
H446	DLBCL	U133+2	GSM2306906
H447	DLBCL	U133+2	GSM2306907
H448	DLBCL	U133+2	GSM2306908
H449	DLBCL	U133+2	GSM2306909
H45	DLBCL	U133+2	GSM2306910
H450	DLBCL	U133+2	GSM2306911
H451	DLBCL	U133+2	GSM2306912
H452	DLBCL	U133+2	GSM2306913
H454	DLBCL	U133+2	GSM2306914
H456	DLBCL	U133+2	GSM2306915
H457	DLBCL	U133+2	GSM2306916
H473	DLBCL	U133+2	GSM2306917
H479	DLBCL	U133+2	GSM2306918
H484	DLBCL	U133+2	GSM2306919
H281	FL	U133+2	GSM2306870
H292	FL	U133+2	GSM2306871
H310	FL	U133+2	GSM2306872
H312	FL	U133+2	GSM2306873
H316	FL	U133+2	GSM2306874
H317	FL	U133+2	GSM2306875
H328	FL	U133+2	GSM2306876
H302	Primary	U133+2	GSM2306877
H302	Primary	SNP6.0	GSM2307498
H385	Relapse	U133+2	GSM2306878
H385	Relapse	SNP6.0	GSM2307499
BB177	Saliva	SNP6.0	GSM2307500

The Affymetrix .CEL files were deposited at the GEO website under GSE86622. In total, U133 plus 2.0 arrays were performed on 41 DLBCL, 2 tFL and 7 FL patient samples and 3 SNP6.0 arrays were performed on the saliva, primary and relapse tumors on the patient case. Clinical data are registered in the National Clinical Quality Database for Malignant Lymphoma (www.lymphoma.dk).
